# Stress in Obesity and Associated Metabolic and Cardiovascular Disorders

**DOI:** 10.6064/2012/205027

**Published:** 2012-12-31

**Authors:** Paul Holvoet

**Affiliations:** Atherosclerosis and Metabolism Unit, Department of Cardiovascular Sciences, KU Leuven, Herestraat 49, P.O. Box 705, 3000 Leuven, Belgium

## Abstract

Obesity has significant implications for healthcare, since it is a major risk factor for both type 2 diabetes and the metabolic syndrome. This syndrome is a common and complex disorder combining obesity, dyslipidemia, hypertension, and insulin resistance. It is associated with high atherosclerotic cardiovascular risk, which can only partially be explained by its components. Therefore, to explain how obesity contributes to the development of metabolic and cardiovascular disorders, more and better insight is required into the effects of personal and environmental stress on disease processes. In this paper, we show that obesity is a chronic inflammatory disease, which has many molecular mechanisms in common with atherosclerosis. Furthermore, we focus on the role of oxidative stress associated with obesity in the development of the metabolic syndrome. We discuss how several stress conditions are related to inflammation and oxidative stress in association with obesity and its complications. We also emphasize the relation between stress conditions and the deregulation of epigenetic control mechanisms by means of microRNAs and show how this impairment further contributes to the development of obesity, closing the vicious circle. Finally, we discuss the limitations of current anti-inflammation and antioxidant therapy to treat obesity.

## 1. Introduction

By 2015, approximately 2.3 billion adults will be overweight and more than 700 million will be obese according to the World Health Organization projections. The United States (US) is currently the biggest single market for weight loss drugs, with around 68 percent of the population either overweight or obese, followed by the United Kingdom and other European countries. In the US alone, over 9 million children and teenagers are obese. Moreover, China, Russia, India, and Brazil could soon begin to surpass Western countries in its obese populations.

**Figure 1:**
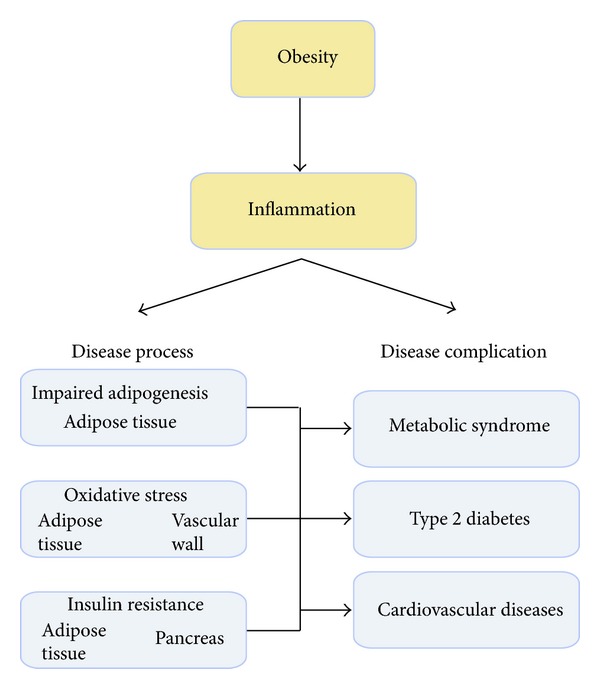
Schematic representation of the central role of inflammation in obesity-induced disorders. The inflammatory state, associated with excessive caloric intake during obesity induces adipose tissue remodelling, oxidative stress, and insulin resistance, associated with an increased risk of developing metabolic syndrome, type 2dibetes, and cardiovascular diseases.

Our first aim is to demonstrate that obesity is a chronic inflammatory disease state that is associated with other disease processes such as adipose tissue (AT) remodelling, oxidative stress, and insulin resistance (IR). These disease processes contribute to the development of type 2 diabetes (T2DM) and the metabolic syndrome (MetS) [1]. MetS is a common and complex disorder combining obesity, dyslipidemia, hypertension, and IR [2–5]. It is a primary risk factor for T2DM and cardiovascular diseases (CVD) [3, 6–13] (Figure 1).

Our second aim is to analyze the effects of behaviour and personal and environmental stress factors on the development of obesity and associated disorders. We emphasize the effects of stress factors on the loss of epigenetic regulatory mechanisms of disease processes, with a primary focus on inflammation.

Finally, we discuss the limitations of current anti-inflammatory and antioxidant treatment of obesity and its associated metabolic and cardiovascular disorders by showing their inappropriate control of disease processes.

## 2. Obesity Is a Chronic Inflammatory Disease

**Figure 2:**
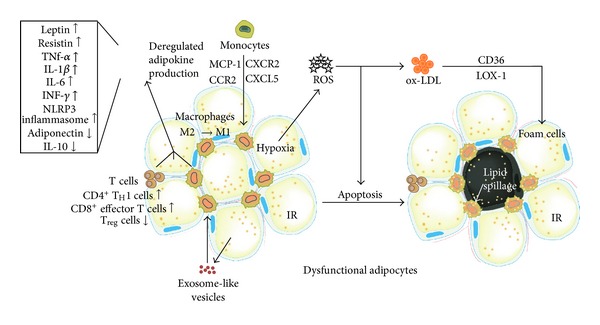
Low-grade chronic inflammation and oxidative stress in adipose tissue during obesity. The excessive accumulation of adipose tissue during obesity is characterized by the recruitment of immune cells. Activated T cells and chemokines induce monocyte migration into adipose tissues where they differentiate into proinflammatory M1 macrophages. The interaction between activated T cells, macrophages, and dysfunctional adipocytes results in a dysregulated adipokine and exosome-like vesicle production causing insulin resistance (IR). Adipose tissue hypoxia during obesity is associated with ROS and ox-LDL production, and foam cell formation. In addition, hypoxia and increased oxidative stress induce apoptosis of adipocytes contributing to insulin resistance.

Low-grade inflammation is a characteristic of the obese state (Figure 2). Circulating concentrations of many inflammatory markers in obese subjects are higher than in lean people, and they are believed to play a role in causing IR and other metabolic disturbances. Inflammatory markers are also higher in ATs of obese people; they are secreted by infiltrating macrophages and by adipocytes themselves. Blood concentrations of inflammatory markers are lowered following weight loss [14]. Both high-glucose and high-fat meals may induce postprandial inflammation. The latter is worsened by advanced glycation end products (AGE) and partly compensated by the inclusion of certain antioxidants or antioxidant-containing foods within the meal [15].

Mechanistically, the cytokine-interleukin- (IL-) 1*β* has emerged as a prominent instigator of the proinflammatory response in obesity [16]. Indeed, lack of Il-1 receptor-I (Il-1RI) protects mice from high-fat diet-induced AT inflammation coincident with improved glucose homeostasis [17]. Another instigator of inflammation is the NLR pyrin domain-containing-3 (Nlrp3, also known as Nalp3 or cryopyrin) inflammasome; its induction is associated with IR. Ablation of Nlrp3 in mice reduced Il-18 and interferon-*γ* (Ifn-*γ*) and improved insulin signalling [18]. The increased secretion of angiopoietin-like protein-2 (Angptl2) by AT also activates an inflammatory cascade and induces chemotaxis of monocytes/macrophages. Angptl2 deletion ameliorated AT inflammation and systemic IR in diet-induced obese mice [19].

In addition, inflammation is caused by changes in levels of adipokines. Overexpression of adipocyte-derived mouse resistin leads to accelerated white AT (WAT) inflammation associated with increased lipolysis and serum-free fatty acids (FA), and IR [20]. In contrast, adipocyte-derived adiponectin protects against inflammation by promoting macrophage polarization toward an anti-inflammatory phenotype [21]. Increases in adiponectin decreased fat content and inflammatory cytokines tumour-necrosis-factor- (Tnf-) *α* and Il-6 in obese rats [22].

Increased infiltration of monocytes and activation into macrophages is another hallmark of obesity. Exosome-like vesicles (ELVs) released from AT activate macrophages leading to IR in mice. ELVs from obese mice were absorbed by peripheral blood monocytes, which then differentiated into activated macrophages with increased secretion of Tnf-*α* and Il-6. Injection of ELVs of obese mice into lean mice resulted in the development of IR. However, when ELVs were intravenously injected into toll-like-receptor- (Tlr-) 4 knockout mice, glucose intolerance and IR were much lower [23]. Mechanistically, the C-C motif-chemokine-receptor- (CCR-) 2/monocyte-chemotactic-protein- (MCP-) 1 (also CCL2) system regulates monocyte and macrophage recruitment. Indeed, short-term treatment with a pharmacological antagonist of Ccr2 lowered macrophage content of AT and improved insulin sensitivity without significantly altering body mass in mice [24]. Furthermore, the chemokine CXC-ligand- (CXCL-) 5 was also increased in the macrophage fraction of WAT and in serum of human obese subjects and decreased after weight reduction. CXCL5 blocks insulin signalling by activating the janus kinase-2/signal transducer and activator of transcription-5/suppressor of cytokine signalling-2 (Jak2/STAT5/SOCS2) pathways. Obese, insulin-resistant mice treated with either anti-CXCL5 neutralizing antibodies or with antagonists of the chemokine (C-X-C motif) receptor (CXCR)-2, which is the CXCL5 receptor, were protected against IR, as were Cxcr2−/− mice [25].

In addition to an increased number of macrophages in obese AT, their phenotype is different. Indeed, whereas early stages of AT expansion are characterized by anti-inflammatory M2-polarized AT macrophages [26], further AT expansion is associated with increases in proinflammatory Cd11c(+)Cd206(+) macrophages that correlate with markers of IR [27]. The profile of macrophages in old fat, independent of further expansion, also shifts toward a proinflammatory environment. The mechanism of this aging-induced shift in the phenotypic profile of macrophages was found to be related to a decrease in peroxisome-proliferator-activated-receptor- (Ppar-) *γ* expression in macrophages and to alterations in Ccr expression profiles [28]. The accumulation of proinflammatory M1 macrophages and the expression of proinflammatory cytokines and chemokines (i.e., Il-6 and Ccl2) was largely blunted in AT of obese mice lacking the leukotriene B4 receptor (Blt)-1 [29]. Drug which decreases the number of M1 macrophages not only reduces inflammation but also increases insulin sensitivity. For example, telmisartan, an angiotensin II type-1 receptor blocker and a PPAR-*γ* agonist, improved IR, decreasing body weight gain, visceral fat weight, and adipocyte size without affecting the amount of energy intake [30]. Treatment with the PPAR agonist pioglitazone also decreased the M1-to-M2 ratio in high-fat diet mice, most likely by increasing the expression of Il-10, an anti-inflammatory Th2 cytokine [31]. Administration of recombinant Il-33 to genetically obese diabetic (ob/ob) mice led to reduced adiposity and fasting glucose and increased glucose tolerance and insulin sensitivity. Il-33 also induced accumulation of Th2 cells in AT and polarization of AT macrophages toward an M2 alternatively activated phenotype (Cd206(+)), a lineage associated with protection against obesity-related metabolic events. Systemic overexpression of Il-10 by an adenovirus vector increased the expression of M2 markers in AT. Moreover, IR is associated with both the number of M1 macrophages and the M1-to-M2 ratio. Increased expression of Il-10 after a high-fat diet might be involved in the increased recruitment of M2 macrophages [31].

Adipocyte death and macrophage-mediated AT remodelling further contributes to inflammatory and metabolic complications of obesity (Figure 2). Increased frequency of adipocyte death in AT of high-fat diet coincided with increases in macrophages expressing F4/80 and Cd11c, Tnf-*α*, and Mcp-1 and IR. More specifically, macrophages in crown-like structures (CLS) surrounding dead adipocytes expressed inflammation markers [32]. Inflamed CLS+ obese subjects displayed higher plasma insulin, homeostasis model assessment (HOMA), triglycerides, glucose, blood pressure, high sensitive C-reactive protein (hs-CRP) and lower HDL-cholesterol, and brachial artery flow-mediated dilation compared with lean subjects. Adipose expression of inflammatory genes including cluster-of-differentiation- (CD-) 68, leptin, matrix-metalloproteinase- (MMP-) 9, CD163, and CD8A were significantly greater and vascular endothelial growth factor (VEGF) was lower in the CLS+ group. In contrast, obese subjects with noninflamed fat exhibited a mixed clinical phenotype with lower IR, reduced proatherogenic gene expression, and preserved vascular function as in lean subjects. In multiple linear regression adjusting for age and sex, CLS status and waist circumference were independent predictors of flow-mediated dilation. These findings supported the concept that factors in addition to absolute weight burden, such as qualitative features of AT, might be important determinants of CVD [33]. Mechanistically, macrophage-induced expression and release of MMP-1 and MMP-3 by human preadipocytes is mediated by IL-1*β* activation [34]. The macrophage-derived apoptosis inhibitor of macrophage (AIM) protein is increased in blood and is incorporated into adipocytes, thereby inducing lipolysis in AT. Such a response is required for the recruitment of macrophages and increased chemokine production in adipocytes via activation of TLR4 [35]. Apoptosis of adipocytes is sufficient to initiate a large influx of macrophages into the remnant fat pads. However, these macrophages are anti-inflammatory M2 macrophages and not M1 cells. Thus, adipocyte death is sufficient to initiate macrophage infiltration, and live adipocytes are required to initiate and/or sustain a proinflammatory response within the infiltrating macrophages in AT [36].

Overall, the contribution of inflammation in AT to the development of obesity is very similar to its contribution to the development of atherosclerosis in arterial tissues [37–39]. The contribution of the phenotypic switch from M2 to M1 macrophages to inflammation and IR is crucial in this. Indeed, the I LIKE HOMe study showed similar monocyte heterogeneity in obesity and subclinical atherosclerosis. Body mass index (BMI) was significantly correlated with carotid intima-media thickness (IMT). High CD16(+) monocyte counts were significantly associated with both higher BMI and increased carotid IMT. Adjustment for CD16(+) monocyte counts weakened the correlation between BMI and carotid IMT, suggesting that the increase in CD16(+) monocyte numbers in obesity may partly explain the association between obesity and IMT [40].

## 3. Obesity Is Related to Metabolic Syndrome

Obesity is often clustered with other cardiovascular risk factors, such as dyslipidemia, hypertension, and hyperglycaemia, in MetS. The Third Report of the National Cholesterol Education Program Expert Panel on Detection, Evaluation, and Treatment of High Blood Cholesterol in Adults (ATPIII) highlights the importance of treating patients with MetS to prevent CVD [2]. It defined MetS components as (1) waist circumference ≥102 cm in men and ≥88 cm in women; (2) fasting triglycerides ≥150 mg/dL (1.70 mmol/L); (3) HDL-cholesterol <40 mg/dL (1.03 mmol/L) in men and <50 mg/dL (1.29 mmol/L) in women; (4) blood pressure ≥130/85 mmHg; (5) fasting-glucose ≥100 mg/dL (5.55 mmol/L). Persons with at least three of these components were defined as having MetS [2–5]. The American Heart Association (AHA) and the National Heart Lung and Blood Institute (NHLBI) slightly modified the ATPIII criteria but did not include abdominal obesity as a required risk factor. Moreover, there was no agreement on the definition of abdominal obesity between the International Diabetes Federation (IDF) and AHA/NHLBI. The IDF recommended that the threshold for waist circumference to define abdominal obesity in people of European origin should be ≥94 cm for men and ≥80 cm for women; the AHA/NHLBI, in contrast, recommended cut points of ≥102 and ≥88 cm, respectively, for the two sexes. Recently, IDF and AHA/NHLBI representatives have attempted to resolve the remaining differences between definitions of MetS. Both sides agreed that abdominal obesity should not be a prerequisite for diagnosis but that it is one of five criteria, so that the presence of any three of five risk factors constitutes a diagnosis of MetS [41]. Because this harmonized definition was published only in 2009, most referenced studies used the IDF or ATPIII definition.

The unadjusted and age-adjusted prevalence of MetS according to ATPIII in 2000 in US were 21.8% and 23.7%, respectively. Prevalence increased from 6.7% among participants aged 20 through 29 years to 43.5% and for participants aged 60 through 69 years. Mexican Americans had the highest age-adjusted prevalence of MetS (31.9%). Age-adjusted prevalence was similar for men (24.0%) and women (23.4%) [2]. The age-adjusted prevalence of MetS among US Asian Indians was 26.9% by the original NCEP/ATPIII criteria, 32.7% by the modified NCEP/ATPIII criteria, and 38.2% by the IDF criteria [42]. In a Family Medicine Centre study in Kingston, Ontario, one in every three patients between 40 and 60 years old met the criteria for MetS [43]. The Prediction of MetS in Adolescence Study showed that birth weight, small head circumference, and parental overweight or obesity in at least one parent predicted future MetS (according to IDF) with a sensitivity of 91% and a specificity of 98% [44].

In the Leicester Ethnic Atherosclerosis and Diabetes Risk (LEADER, UK) Study cohort (71.4% white European, 28.6% South Asian; aged 40–75 years), the prevalence of MetS was 29.9% (29.2% South Asian, 30.2% white European) according to ATPIII and 34.4% (34.2% south Asian, 34.5% white European) according to IDF, respectively. With the ATPIII definition, waist circumference was significantly more predictive of MetS than BMI or waist-hip ratio [45]. The Guangdong Nutrition and Health Survey 2002 showed that 7.3% of residents aged 20 and above had MetS, amounting to 4 million residents in southern China. MetS prevalence was higher in the urban population than in the rural population (10.6 versus 4.3%), and women more often had the syndrome than men (8.9 versus 5.2%) [46].

## 4. Oxidative Stress In Obesity Is Associated with the Development of the Metabolic Syndrome

We determined the longitudinal association of oxidized LDL (ox-LDL) and incident of MetS in 1,889 participants of the Cardiovascular Risk Development in Young Adults (CARDIA) Study [47, 48]. The studied CARDIA sample was balanced by age (45% aged 33–39 years, 55% aged 40–45 years), race (52% African-American, 48% white), gender (46% men, 54% women), and education (40% having completed ≤ 12 years of education, 60% having completed > 12 years). Elevated ox-LDL, but not elevated LDL-cholesterol, was associated with a higher risk of future MetS. Elevated ox-LDL was especially associated with the incidence of abdominal obesity, hyperglycaemia, and hypertriglyceridemia [49]. 

A possible explanation for the relation between ox-LDL and obesity is that ox-LDL may be associated with the increase of AT, in agreement with experimental findings that ox-LDL induces adipocyte proliferation either directly [50] or indirectly by increasing the infiltration of inflammatory monocytes/macrophages [51]. The increase in AT mass may also be explained by cellular hypertrophy due to the increased lipid accumulation in the preexisting adipocytes rather than an increase in cell number or differentiation. Indeed, ox-LDL increased triglyceride production by inducing the expression of lipoprotein lipase (LPL) [52] and by inducing the accumulation of FA in adipocytes [53]. Ox-LDL was also found to decrease the production of adiponectin which in contrast with other adipokines is reduced in obese persons, and which suppresses excess reactive oxygen species (ROS) production under high-glucose conditions. This effect has implications for vascular protection in diabetes [54]. Ox-LDL activated c-Jun N-terminal kinase (JNK) and disrupted insulin signalling in both adipocytes and macrophages in a CD36-dependent manner. Macrophages isolated from Cd36(−/−) mice after high-fat diet feeding elicited less JNK activation and inhibition of insulin signalling in adipocytes after coculture compared with wildtype macrophages [55]. Not only CD36 but also LOX-1 was independently associated with IR in AT. LOX-1 expression was increased when macrophages were incubated with an adiponectin-neutralizing antibody [56]. The observed relation between obesity and ox-LDL and that between ox-LDL and MetS is important to understand the association between obesity and MetS [57]. In addition, we demonstrated in the Health Aging, and Body Composition (Health ABC) cohort, comprising 3,033 participants aged 70–79 years, that those with high ox-LDL had a 2.0-fold higher adjusted risk of myocardial infarction even after adjusting for MetS [58]. 

Increases in ox-LDL in association with MetS and atherosclerosis may be due to loss of antioxidant capacity caused by low serum activity of the antioxidant enzyme superoxide dismutase (SOD) [59] or low HDL-associated paraoxonase (PON) antioxidant activity [58]. Increases in ox-LDL could also be due to increased oxidant capacity, for example, by augmented expression of NADPH oxidase (NOX). Indeed, production of reactive oxygen species (ROS) increased selectively in AT of obese mice caused by higher expression of Nox and decreased expression of antioxidant SOD. In cultured adipocytes, elevated FA increased oxidative stress via Nox activation, and oxidative stress decreased production of adiponectin and increased Il-6 and Mcp-1. Finally, in obese mice, treatment with a Nox inhibitor reduced ROS in AT, decreased inflammatory adipokines, and reduced diabetes, hyperlipidemia, and hepatic steatosis [60].

## 5. Metabolic Syndrome Is Associated with Cardiovascular Diseases

MetS is associated with a high risk of CVD. This association is partly dependent on and partly independent of obesity and T2DM. The Finish Kuopio Ischemic Heart Disease Risk Factor Study showed that CVD and all-cause mortality were increased in men aged 42 to 60 years with MetS, even in the absence of baseline CVD and diabetes [9]. Placebo data from the Scandinavian Simvastatin Survival Study (4S) and the Air Force/Texas Coronary Atherosclerosis Prevention Study (AFCAPS/TexCAPS) were used post hoc to estimate the long-term relative risk of major coronary events (MCEs) associated with MetS, after excluding diabetes mellitus. In 4S and AFCAPS/TexCAPS, respectively, placebo-treated patients with MetS were one and a half times more likely to have MCEs than those without it. Of the MetS components, low HDL-cholesterol was associated with elevated risk of MCEs in both studies, whereas high triglycerides in 4S and elevated blood pressure and obesity in AFCAPS/TexCAPS were associated with significantly increased relative risk. Patients with MetS showed increased risk of MCEs irrespective of their Framingham-calculated 10-year risk score category. These data suggested that MetS is associated with risk that is not entirely accounted for by traditional risk scoring [6]. In the Second National Health and Nutrition Examination Survey, age-, gender-, and risk factor-adjusted hazard ratios (HRs) for coronary heart disease (CHD) mortality were twice higher for those with MetS and four-fold higher for those with preexisting CVD. In persons with MetS but without diabetes, risks of CHD and CVD mortality remained elevated. Diabetes predicted all mortality end points [11]. We studied the impact of MetS (38% prevalence) on outcomes in 3,035 participants in the Health ABC study (51% women, 42% black, ages 70 to 79 years). After adjusting for baseline characteristics, patients with MetS were at a significantly higher risk of coronary events, myocardial infarctions, and heart failure. The coronary event rate was higher among subjects with diabetes and with MetS. Subjects with both diabetes and MetS had the highest risk [61]. At Mayo Clinic, a positive gradient for CHD, CVD, and all-cause mortality rates across exercise electrocardiographic (E-ECG) categories with three, four, or five MetS components was observed in 9,191 men. These findings underscored the importance of E-ECG tests to identify men with MetS who are at risk of dying [62]. Development of cardiac allograft vasculopathy (CAV) was significantly higher in patients with MetS who had undergone heart transplantation (59% versus 19%). Patients with a higher score of MetS criteria had a higher risk of CAV: no criteria (4%); one criterion (4%); two criteria (47%); three criteria (62%); four criteria (75%); and five criteria (100%) [63]. 

Grundy and colleagues measured a series of cardiovascular risk factors in 59,820 men and 22,192 women. The risk factor profiles were segregated into five quintiles of cardiorespiratory fitness (CRF). With decreasing CRF, increases occurred in obesity, triglycerides, triglyceride/HDL lipoprotein ratios, blood pressure, MetS and diabetes, and smoking [64]. In the Taiwan Survey of Hypertension, Hyperglycaemia and Hyperlipidemia cohort, the MetS-attributed risk for CVD was 39% in men and 44% in women. Of all MetS components, central obesity had the highest population attributable risk (PAR) in women (57%) whereas hypertension had the highest PAR in men (57%) [65]. In Jiangsu, China, MetS was associated with a more than two-fold higher CVD risk after adjustment for age, sex, gender, BMI, alcohol consumption, family history of CVD, and smoking [66]. 

Juonala et al. performed a meta-analysis of data from four prospective studies to determine whether this risk is reduced in persons who are overweight or obese as children but not as adults. The mean length of followup was twenty-three years. Subjects with consistently high adiposity status from childhood to adulthood had a five-fold higher risk of T2DM, and a two-fold higher risk of hypertension and of lower HDL-cholesterol, associated with an increased IMT of the carotid artery. Interestingly, the risks of these outcomes among overweight or obese children who became nonobese by adulthood were similar to those among persons who had never been obese [67].

## 6. Behaviour and Obesity

**Figure 3:**
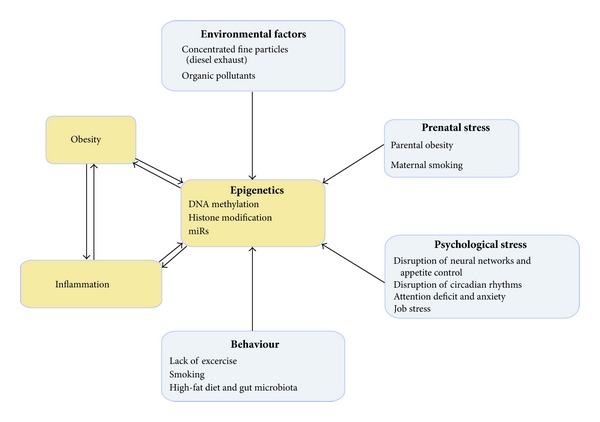
The interaction between different stress factors and pathophysiological processes in obesity. Behaviour and physiological and environmental stress conditions impair epigenetic control mechanisms resulting in exacerbation of inflammation that is already increased in adipocytes and circulatory and tissue inflammatory cells in obese subjects.

There is growing evidence that the lack of physical exercise, smoking, and fat rich diets contribute to the development of obesity and associated disorders (Figure 3).

### 6.1. Exercise

Dose-response relationships between exercise and metabolic risk have been demonstrated in adults [68] and in children [69, 70]. The Diabetes Prevention Program demonstrated that diet and exercise reduced the risk of diabetes in adults with prediabetes [71]. A 2003–2007 randomized controlled efficacy trial in overweight or obese sedentary children (mean age, 9.4 years; 42% male; 58% black) recruited from fifteen public schools showed that after thirteen weeks, 20 or 40 minutes/day of aerobic training improved fitness and demonstrated dose-response benefits for IR and general and visceral adiposity, regardless of sex or race [72]. Interventions that (i) combined high levels of parental involvement and interactive school-based learning, (ii) targeted physical activity and dietary change, and (iii) included long-term followup appeared most effective in preschool- and school-based obesity prevention [73].

Regular physical exercise contributes to diminishing total, visceral, and subcutaneous fat, even without weight loss, as well as to reduced glycaemia and to increased free FA (FFA) oxidation and, thus, to an amelioration of IR [74]. Exercise can indeed be considered as an “insulin-like” activity because of the muscle's increased capacity to capture circulating glucose, a result from decreased intramuscular fat reserves [74]. Mechanistically, exercise training significantly increased the expression of the glucose-transporter- (GLUT-) 4 [75] and of the insulin-receptor-substrate- (IRS-) 1 and the posttranscriptional regulation of the PI3-kinase expression [76]. Among other benefits, exercise stimulates lipolytic activity (with decreased plasma triglycerides), promotes the use of FFA as an energy source, and increases HDL concentration. Furthermore, it increases the antioxidant PON activity of HDL [77]. Adaptation to oxidative stress in trained individuals is clearly evidenced by increased antioxidant protection and by increased resistance against chronic ROS production [78]. In addition, exercise seems to reduce low-grade chronic inflammation, albeit this reduction may depend on the decrease of glycated haemoglobin (HbA1c), fasting glucose, and fat mass [79, 80]. The increase in the antioxidant and anti-inflammatory capacity may be due to an increase in adiponectin [81].

Data from the Aerobics Center Longitudinal Study show that low cardiovascular fitness accounted for almost all of the excess all-cause mortality among obese men [82]. After adjustment for age, examination year, cigarette smoking, alcohol intake, and parental history of ischemic heart disease, unfit (low CRF as determined by maximal exercise testing), lean men had twice the risk of all-cause mortality of fit, lean men. Unfit, lean men also had a higher risk of all-cause and CVD mortality than did men who were fit and obese. Similar results for fat and fat-free mass in relation to mortality were observed. Unfit men had a higher risk of all-cause and CVD mortality than did fit men in all fat and fat-free mass categories. Similarly, unfit men with low waist girths (<87 cm) had a greater risk of all-cause mortality than did fit men with high waist girths (≥99 cm). Three times more obese women who met current physical activity guidelines had a healthy metabolic profile than those who did not meet current physical activity guidelines. These data suggest that increasing physical activity may reduce risk even in those who do not succeed in reducing their BMI.

In two prospective cohorts with 7,740 women and 4,564 men, a genetic risk score based on 32 established BMI-associated variants to capture the overall genetic susceptibility was calculated, and its interactions with leisure time TV watching and physical activity in relation to adiposity were determined. Prolonged TV watching was found to accentuate the genetic predisposition to elevated adiposity, whereas greater leisure time physical activity attenuated the genetic association. These findings suggested that deleterious effects of genetic factors could be modified by lifestyle factors and challenge the common lay perception of deterministic genetic predisposition to obesity [83]. 

### 6.2. Smoking

In the short term, nicotine increases energy expenditure and could reduce appetite, which may explain why smokers tend to have lower body weight than do nonsmokers and why smoking cessation is frequently followed by weight gain. In contrast, heavy smokers tend to have greater body weight than do light smokers or nonsmokers, likely reflecting a clustering of risky behaviours (e.g., low degree of physical activity, poor diet, and smoking) that is conducive to weight gain. The Ottawa Hospital Weight Management study showed that obese former smokers have a greater prevalence of impaired glucose, T2DM, and coronary heart disease than obese subjects who had never smoked [84]. In the context of the worldwide obesity epidemic and a high prevalence of smoking, the greater risk of (central) obesity and IR among smokers is thus a matter of major concern. Furthermore, smoking was found to increase the number and level of oxidation products of phospholipids (oxPAPC) in peripheral blood mononuclear cells causing ROS generation via NOX activation due to reduced glutathione. Smoking also activated NF-*κ*B and increased hs-CRP values [85]. 

### 6.3. High-Fat Diet and Gut Microbiota

Studies in obese and lean twins suggest that a core gut microbiome exists and that obese persons exhibit reduced diversity and have altered metabolic pathways in their microbiota. Diet may have a fundamental effect on the composition of our microbiota. Early studies highlight the effect of specific diets such as a high-fat diet, which efficiently and very rapidly (within a single day) modulates the gut microbiome [86]. Overall, the qualitative and quantitative changes in the intake of specific food components (FA, carbohydrates, micronutrients, prebiotics, probiotics) do not only have consequence for gut microbiota composition but may modulate the expression of genes in host tissues such as liver, AT, intestine, and muscle. This in turn may stimulate or reduce the development of fat mass and metabolic disturbances associated with the gut barrier function and with systemic immunity. Recent work has shown that gut bacteria can initiate the inflammatory state of obesity and IR through the activity of lipopolysaccharide (LPS), a component of the gram-negative bacteria cell walls, which can trigger the inflammatory process by binding to the CD14-TLR4 complex. The relevance of TLR4 pathways for metabolic disease was confirmed by the finding that the deletion of Tlr4 prevented high-fat diet-induced IR [87]. Moreover, Cd14 knockout rats showing reduced inflammatory reaction to LPS were immune to weight gain [88]. 

Especially a reduction in Lactobacilli and Bifidobacteria may affect host metabolism and the inflammatory state by modulating the tissue FA composition. Indeed, mammalian intestinal Lactobacilli and Bifidobacteria can synthesize bioactive isomers of conjugated linoleic acid from free linoleic acid, which have antidiabetic, antiatherosclerotic, immunemodulatory, and anti-obesity properties (for review: [89]). The supplementation of Bifidobacterium breve and linoleic acid to different mammalian species compared to a linoleic acid-alone supplemented diet resulted in a two- to three-fold higher intestinal, hepatic, and AT content of *cis*-9, *trans-*11 conjugated linoleic acid, eicosapentaenoic acid, and docosahexaenoic acid, concomitantly with a reduced proinflammatory cytokines Tnf-*α*, Il-6, and Inf-*γ* expression, [90]. In mice, supplementation with *Lactobacillus reuteri* 100-23 and *Lactobacillus gasseri* 311476 decreased inflammatory cytokines (Il-6, Mcp-1, Il-4, and granulocyte colony-stimulating factor). These positive effects are strain and/or species specific since *L. acidophilus* NCFM supplementation has no effect on muscle atrophy markers and systemic inflammation [91].

## 7. Psychological Stress and Obesity

Psychological stress can have a significant long-term impact on the propensity to gain and maintain weight (Figure 3). The interrelatedness between obesity and psychological problems seem to be two-fold, in that clinically meaningful psychological distress might foster weight gain and obesity may lead to psychosocial problems, closing a vicious circle. A recent study investigated the relationship between severity of 15 stressful life events pertaining to finance, work, social relationships, health, and housing, and the risk of metabolic disorders. In comparison with subjects who did not report any extremely stressful life events, those reporting work- or finance-related events had a higher risk of having MetS. The risk was further increased by the accumulation of stressful finance-related events. Accumulation of stressful life events was associated with IR, obesity, and raised triglycerides. The associations were not confounded by sex, age, lifestyle, or a family history of diabetes. It was concluded that life events perceived as stressful, particularly those related to finance and work, may signal poor metabolic health [92].

### 7.1. Disruption of Neural Networks and Appetite Control

Obesity, particularly the abdominal phenotype, has been ascribed to individual maladaptation to chronic stress exposure mediated by a dysregulation of related neuroendocrine axes. Alterations in the control and action of the hypothalamic-pituitary-adrenal axis play a major role in this context, with the participation of the sympathetic nervous system [93]. Especially the inability to cope with psychological stress, particularly early life stress, ultimately leads to an increase in glucocorticoids, which in turn leads to loss of appetite control and increased adiposity [94]. 

In addition, neuroimaging studies showed that obesity is associated with impaired reward and tolerance processes. Indeed, many obese individuals meet criteria for psychological dependence. Stress and dieting may sensitize an individual to reward. Finally, fast food advertisements, restaurants, and menus all provide environmental cues that may trigger addictive overeating. While the concept of fast food addiction remains to be proven, some findings support the role of fast food as a potentially addictive substance, which is most likely to create dependence in vulnerable populations [95]. Therefore, it has been proposed that in order to increase prevention and intervention efforts with regard to childhood obesity, early detection of psychological factors contributing to its development and maintenance is required. Rather than a stable condition, childhood obesity is a dynamic process, in which behaviour, cognition, and emotional regulation interact mutually with each other. Family structure and context, that is, parental and familial attitudes, activity and nutritional patterns as well as familial stress, have an important role with respect to both onset and maintenance of overweight and obesity. 

### 7.2. Disruption of Circadian Rhythms

The circadian system is tightly linked with processes controlling not only sleep but also metabolism. These dynamic interactions ensure that the energy metabolism is coordinated in a proper temporal pattern and that circadian control is also subject to modulation by the energy status of the organism. Disruption of either the circadian clock or metabolism can lead to derangement of the other, thus predisposing to metabolic disorders such as obesity and T2DM (for review: [96]). High-fat diets disrupt circadian mechanisms in mouse AT and these effects appear to be due to obesity rather than to hyperglycaemia. Deletion of circadian regulatory genes such as 5′-AMP-activated-protein-kinase- (AMPK-) 1 and Nocturnin alter the circadian biology of AT. Neuroendocrine and behavioural studies have demonstrated a potential circadian arrhythmicity among those with night-eating syndrome (NES), which is characterized by increased late-night eating, insomnia, depressed mood, and distress. Several physiological systems have been hypothesized to be involved in the mechanistic drive in NES, such as the glucocorticoid and serotonergic systems. This circadian arrhythmicity could be one of the links between NES and obesity, as emerging findings have linked chronodisruption with increased body weight (for review: [97]). In addition, studies published in the past 10 years tend to document an impact of shift work-related disturbances of circadian clock on blood pressure, lipid profile (triglyceride levels), MetS, and BMI (for review: [98]), but underlying mechanisms remain to be determined. 

### 7.3. Attention Deficit and Anxiety

Behavioural and emotional problems are found in many, though not all, obese children, resulting in a higher need of treatment. The most frequently implicated psychosocial factors are impulsivity and attention-deficit hyperactivity disorder, depression and anxiety, and uncontrolled eating behaviour. These findings strengthen the need to further explore the interrelatedness between psychological problems and childhood obesity [99]. In addition, recent studies demonstrated that these stressors are particularly prevalent in children from low-income families, a demographic group with high rates of obesity in the US and other developed countries. Therefore, policy recommendations emerging from this research included recognizing reductions in childhood obesity as a potential added benefit of social safety net programs that reduce financial stress among families. In addition, policies and programs geared towards childhood obesity prevention should focus on helping children build resources and capacities to teach them how to cope effectively with stressor exposure (for review: [100]). Unfortunately, the effectiveness of paediatric obesity treatment is still modest in younger children and even declines in older children and adolescents. Moreover, few interventions involving adolescents have produced a significant long-term weight loss. It has been concluded that a key parenting practice applicable to children of all ages is to create a protective environment in the home, substituting unhealthy foods by nutritious to unhealthy ones and facilitating physical activities instead of sedentary pursuits. Other behaviour that may promote successful long-term weight management include good sleep hygiene and stress reduction [101]. Although broader changes to the food environment are necessary, it is important to address personal factors such as nutrition knowledge, self-sufficiency, and emotional coping responses to stress, in the context of income constraints, food insecurity, and health beliefs [102, 103].

### 7.4. Job Stress

Because data about the association between job stress and obesity were inconsistent, mostly limited to small-scale studies, and did not distinguish between categories of underweight or obesity, Nyberg et al. performed a pooled cross-sectional analysis based on 746 individual-level data from 13 European studies resulting in a total of 161 years. In cross-sectional analyses, they found increased odds of job strain amongst underweight and obese participants. In longitudinal analysis, both weight gain and weight loss were related to the onset of job stress, consistent with a “U”-shaped cross-sectional association between job strain and BMI. However, these associations were relatively modest; therefore, it was concluded that it is unlikely that intervention to reduce job stress would be effective in combating obesity at a population level [104].

## 8. Prenatal Stress

There is growing evidence that stress factors imposed on the foetus increase risk of obesity and associated metabolic disorders (Figure 3). For example, the Quebec Longitudinal Study of Child Development 1998–2002 (QLSCD) examined a broad range of factors that may simultaneously contribute to childhood overweight in a population-based cohort of children followed from birth to 4.5 years, to determine which factors exert the most influence in early life. Maternal smoking during pregnancy almost doubled the odds of being overweight at 4.5 years. Parental overweight or obesity also increased the odds of being overweight at this age, as well as being raised in middle-income or in poor families [105]. Proteomic analysis of placenta shows a differential expression of several proteins in patients with preexisting obesity. These proteins are implicated in a variety of cellular functions such as regulation of growth, cytoskeletal structure, oxidative stress, inflammation, coagulation, and apoptosis [106]. On the other hand, children that were nutrient-restricted in utero were shown to have increased adipocyte sensitivity to cortisol. This adaptation only appears to be associated with greater fat mass in children when maternal nutrient restriction is confined to late gestation, coincident with the period of maximal foetal growth. In these children, increased fat mass is accompanied by glucose intolerance and IR, in conjunction with an adipose tissue specific reduction in GLUT-4 [107]. Animal studies revealed that maternal low-protein diet upregulates the neuropeptide Y system in visceral fat and leads to abdominal obesity and glucose intolerance in a similar way as does the stress hormone epinephrine [108, 109]. A maternal “junk food” diet in pregnancy and lactation was associated with increased de novo lipogenesis, lipid oxidation, inflammation, and IR in offspring [110].

## 9. Environmental Stress and Obesity

Elucidating the environmental factors that influence susceptibility to disruptions in energy homeostasis and metabolic regulation remains a challenge. We confined this paper to two pollutants which were found to be associated with inflammation and metabolic disorders (Figure 3).

### 9.1. Particulate Matter and Diesel Exhaust

There is a strong link between urbanization and T2DM. Therefore, it has been proposed that ambient air pollutants may play a role in the development of T2DM. This hypothesis was tested in mice. Male C57BL/6 mice were fed high-fat chow for 10 weeks and randomly assigned to an environment with concentrated fine particulate matter (<2.5 *μ*m; particulate matter (PM)(2.5)) or filtered air. PM(2.5)-exposed C57BL/6 mice exhibited marked whole-body IR, systemic inflammation, and an increase in visceral adiposity. PM(2.5) exposure induced signalling abnormalities characteristic of IR, including decreased Akt and endothelial nitric oxide synthase (eNOS) phosphorylation in the endothelium and increased protein kinase C expression. In addition, PM(2.5) increased AT macrophages (F4/80(+) cells) in visceral fat expressing higher levels of Tnf-*α*/Il-6 and lower Il-10/N-acetyl-galactosamine specific lectin-1 [111]. Moreover, the effect of early life exposure to PM(2.5) pollution on metabolic parameters, adiposity, and inflammation and oxidative stress was tested. PM(2.5)-exposed C57BL/6 mice fed a normal diet exhibited metabolic abnormalities after exposure to PM(2.5) or FA for 10 weeks. Consistent with IR, these abnormalities included enlarged subcutaneous and visceral fat contents, increased macrophage infiltration in visceral AT, and vascular dysfunction. Ex vivo labelled and infused monocytes demonstrated increased adherence in the microcirculation of normal diet- or high-fat diet-fed PM(2.5)-exposed mice. p47(phox−/−) mice exhibited an improvement in parameters of IR, vascular function, and visceral inflammation in response to PM(2.5). ROS generation by NOX appeared to mediate this risk [112]. Interestingly, the relationship between diesel exhaust particles and oxidative stress was confirmed in a cross-sectional study, suggesting their proatherogenic nature [113]. 

### 9.2. Organic Pollutants

Evidence also points to endocrine disrupting chemicals, that is, persistent organic pollutants (POPs) such as tributyltin (TBT) and triphenyltin (TPT), which interfere with AT biology, endocrine hormone systems, or central hypothalamic-pituitary-adrenal axis. They are thus suspected to derail the homeostatic mechanisms important to weight control [114]. For example, TBT was found to drive the differentiation of adipocytes and it modulates the retinoid-X-receptor- (RXR-) PPAR-*γ*-dependent proadipogenic gene networks in liver, AT, and bone marrow [115–117]. It appears that RXR heterodimeric nuclear receptors provide cells with a coordinated and interrelated network of transcriptional regulators for interpreting the lipid milieu and regulating metabolic changes to respond to it [118]. 

What is yet to be explored in detail is a disease model based on long-term effects of low doses of environmental exposures, and on the cumulative effects of different exposures. Therefore, recent developments of “-omic” high-throughput technologies, such as transcriptomics, proteomics, and metabolomics, may provide powerful tools to investigate early effects of environmental exposures and to understand the aetiology of common diseases better, according to the “clinical vulnerability model” [119]. For example, prenatal exposure to tobacco smoke increases the risk of CVD later in the child's life; these effects could in part be mediated by epigenetic changes in children exposed prenatally to tobacco smoke [120]. Similarly, Baccarelli et al. showed that pollution from traffic, an environmental challenge associated with increased CVD risk, affected DNA methylation [121].

## 10. Epigenetic Regulation

Researchers are increasingly exploring the epigenome, which is the malleable interface of gene-environment interactions. Epigenetic variation, whether innate or induced, contributes to variation in gene expression, the range of potential individual responses to internal and external cues, and risk for metabolic disease [122]. It is now accepted that obesity and associated metabolic disorders arise from a set of complex gene-environment interactions. Explanations for the heritability of these syndromes and the environmental contribution to disease susceptibility are addressed by the “thrifty genotype” and the “thrifty phenotype” hypotheses. Recently, Stöger synthesized a “thrifty epigenotype” hypothesis as follows: (i) metabolic thrift, the capacity for efficient acquisition, storage, and use of energy, is an ancient, complex trait, (ii) the environmentally responsive gene network encoding this trait is subject to genetic canalization and has become robust against mutational perturbations, (iii) DNA sequence polymorphisms play a minor role in the aetiology of obesity and T2DM. Instead, disease susceptibility is predominantly determined by epigenetic variations, (iv) corresponding epigenotypes have the potential to be inherited across generations, and (v) leptin is a candidate gene for the acquisition of a thrifty epigenotype [123]. 

It has been suggested that hormonal and metabolic signals acting during the perinatal period alter the structure and function of the fat-brain axis or adipogenic genes, such as leptin and PPARs, in AT that regulates energy during later life [124, 125]. Alternatively, it has been proposed that intrauterine exposures may produce long-term changes in mRNA levels leading to a thrifty phenotype with changes affecting liver and muscle physiology [126] as well as long-lasting changes that can be associated with obesity and IR [127], and atherosclerosis and heart failure in later life. Compelling data suggested a strong causal link between prenatal vulnerability of future parental epigenomes to damaging environmental factors aggravated by abnormal sociocultural conditions (including, for instance, malnutrition and chronic stress) and the alarming risk of developing heritable complex medical conditions later in life, such as asthma, autism, cancer, CVD, diabetes, obesity, schizophrenia, and a whole range of rare neuromuscular pathologies [128]. Increasing evidence shows that environmental and lifestyle factors may influence epigenetic mechanisms, such as DNA methylation, histone acetylation and microRNA (miR) expression (Figure 3). It has been shown that several lifestyle factors such as diet, obesity, physical activity, tobacco smoking, alcohol consumption, environmental pollutants, psychological stress, and working night shifts might modify epigenetic patterns. Among them, epigenetically mediated signal-specific inflammatory mechanisms may be involved. They may operate through transcription factors (NF-*κ*B family), kinases (I*κ*B kinase-related kinases, serine/threonine protein kinase), the endoplasmatic reticulum (Ca), activation of DNA methyltransferases and histone modifier enzymes (histamine methyl transferase), and changes in cellular acetyl-CoA, nicotinamide adenine dinucleotide (NAD), and methyl donors. They are all sensitive to oxidative stress, hyperglycaemia, and FA loads [129, 130]. The finding that loss of function of the histone demethylase, Jhdm2a, was associated with obesity, decreased expression of metabolically active genes (e.g., Ppar-*α* and medium-chain acyl-CoA dehydrogenase) in skeletal muscle, and an impaired cold-induced uncoupling-protein- (Ucp-) 1 expression in brown AT (BAT) in rodents also suggested a relationship between epigenetic mechanisms and obesity [131, 132]. In addition, a deregulation in the NAD(+)-dependent sirtuins (SIRT), especially Sirt1, is associated with disruptions in adipogenesis, mitochondrial biogenesis, glucose utilization, fat oxidation, and insulin secretion in mice [133]. The circadian activity of the deacetylase SIRT1 is regulated by NAD, which constitutes a link between cyclic cellular metabolism and epigenetic regulation by chromatin remodelling [134, 135]. 

Most of the epigenetic studies conducted so far have focussed on DNA methylation, whereas only a few have studied lifestyle factors in relation to histone modifications and miRs [136]. MiRs are highly conserved noncoding RNA molecules of approximately 22 nucleotides that exert posttranscriptional effects on gene expression. They generally bind to target sequences localized in the 3′-untranslated region (3′-UTR) of their target mRNAs and regulate protein translation or mRNA stability (for review: [137, 138]). Early studies in *Caenorhabditis elegans* determined that miR-mediated regulation was posttranscriptional, because there were large effects on protein expression and no discernible effects on mRNA abundance [139]. In other systems, modest effects on the amounts of mRNA target were seen in addition to substantial degrees of regulation at the protein level [140]. Other studies provided experimental support for the proposed postinitiation mechanism of translational repression by miRs [141]. MiRs are expressed in a tissue- and cell-type specific manner and play essential roles in many biological processes including proliferation, apoptosis, development, and differentiation [142, 143]. Recently, we reviewed miRs that play an active role in the development of obesity and atherosclerosis [38]. Therefore, this paper will include only a limited number of examples of miRs related to stress conditions in obesity.

### 10.1. MiRs Linking Stress with Inflammation

Dicer, an RNase III type endonuclease, is required for biogenesis of miRs and small interfering RNAs (siRNAs) and also has an important role in an effector step of RNA silencing, the RNA-induced silencing complex (RISC) assembly [144]. Dicer ablation, resulting in impaired miR production in the central amygdale of adult mice induced a strong increase in anxiety-like behaviour. In addition, acute stress in wildtype mice induced a differential expression profile of miRs in the amygdale. For example, miR-34c that targets the stress-related corticotropin-releasing-factor-receptor- (CRFR-) 1 mRNA reduced the responsiveness of cells to CRF in neuronal cells endogenously expressing CRFR1 *in vitro*. Thus, these results suggested a physiological role for miRs in regulating the central stress response and position them as potential targets for the treatment of stress-related disorders [145]. In addition, miRs have recently been shown to be differentially expressed in brain tissue and have been linked to the regulation of neural factors specific to obesity, in particular the control of appetite, and in neural signalling to liver, muscle, pancreas, and gastrointestinal tract, to influence metabolism (for review: [146]). SIRT1-related miR-132 has been shown to be highly expressed in brain tissue and neuronal cell types and was found to be involved in the regulation of cAMP response element-binding protein (CREB), which is also known to function in glucose homeostasis [147]. Interestingly, miR-132 potentiated cholinergic anti-inflammatory signalling by targeting acetylcholinesterase, suggesting that miR-132 is a functional regulator of the brain-to-body resolution of inflammation [148]. Moreover, several miRs are commonly overexpressed in both brain and pancreatic *β*-cells, suggesting an overlap in function (e.g., miR-9, miR-124a). Overexpression of the SIRT1 related miR-9, previously thought to be a brain-specific miR, in insulin-secreting cells caused a reduction in insulin exocytosis by diminishing the expression of the transcription factor Onecut-2 and, in turn, by increasing the level of Granuphilin/Slp4, a Rab GTPase effector associated with *β*-cell secretory granules, which exerts a negative control on insulin release [149]. 

Importantly, miR-9 is involved in the regulation of inflammation because TLR4-activated NF-*κ*B rapidly increased the expression of miR-9, which operated as a feedback of the NF-*κ*B-dependent responses [150]. Similarly, miR*-*124a was initially found to be overexpressed in brain and neural tissue and was subsequently found to be abundant in pancreatic *β* cells [151]. There, it targets FoxA2, also known as HNF3*β*, a transcription factor important for *β*-cell differentiation, pancreatic development, glucose metabolism, and insulin secretion [152].

### 10.2. MiRs Linking Physical Activity with Inflammation

In peripheral human monocytes, exercise decreased the expression level of miRs important in inflammatory processes (e.g., miR-125b) in general and TLR4 signalling (e.g., let-7e) in particular. 

### 10.3. MiRs Linking Circadian Rhythm with Adipogenesis

Recently links between circadian regulators, chromatin remodelling, and cellular metabolism have been identified [153]. In addition, some studies have suggested that miRs (e.g., miR-96, miR-103, miR-106, miR-124, miR-132, miR-182, miR-219, miR-263, and miR-422) may be important regulators of circadian rhythm, which provides a new insights into biological clocks at the posttranscriptional level (for review: [154]). Interestingly, functional studies with synthetic miR precursors and inhibitors showed that miR-96 and miR-124 regulated the expression of fatty-acid-binding-protein- (FABP-) 4, a prominent regulator of adipogenesis [155], in bone-marrow derived mesenchymal cells [156], which are precursors of adipocytes [157]. The miR-182/96/183 cluster was shown to be important for PPAR-mediated adipogenesis [158]. The expression of miR-132 correlated with BMI, fasting glucose, and glycosylated haemoglobin in obese persons [159].

### 10.4. MiRs Linking Gut Microbiota with Circadian Clock, Inflammation, and Insulin Signalling

It has been suggested that caecal miR expression signature depends on the presence of gut microbiota [160]. Germ-free and conventionally raised mice were used to investigate the impact of endogenous microbiota on the global expression of caecal miRs *in vivo.* Among the most differentially expressed miRs were miR-143, miR-192, miR-200b, miR-200c, and miR-24 [161]. MiR-143 overexpression accelerated adipogenesis *in vitro* [162] and induced obesity-associated diabetes in mice [163]. The miR-192/194 cluster regulated the Period gene family and the circadian clock [164]. The miR-200 family was found to be proinflammatory [165]. MiR-24 negatively regulated the expression of forkhead-box- (FOX-) P3, which is essential for the development and function of  T_reg_ cells [166].

### 10.5. MiRs and Air Pollution

Exposure to ambient PM(2.5) altered the expression of two inflammation-related miRs, that is, miR-21 and miR-222 [167]. The expression of miR-221 and miR-222 depends on dietary conjugated linoleic acid in mice and correlated with the expression of adipokines [168]. Exposure of bronchial epithelial cells, obtained from nonsmoking adult donors, to diesel exhaust particles *in vitro *altered the expression of 313 miRs, among them miR-513a-5p, miR-494, and miR-96, which target genes involved in inflammation pathways (e.g., IL-8 and CXCR4 signalling) [169, 170]. 

In aggregate, these data support a model in which lack of physical activity, disruption of circadian rhythm, and psychological and environmental stress deregulates the expression of miRs. This deregulation disrupts adipogenesis, either directly or indirectly through inflammation, and contributes to the development of obesity and associated disorders. 

## 11. Anti-Inflammatory and Antioxidant Therapy in Obesity

Although obesity is considered to be a chronic inflammatory disease, there are no long-term large-scale intervention studies showing that anti-inflammatory drugs can be safely used to treat obesity and associated disorders. For example, salsalate (nonacetylated salicylate, 4500 mg/day), a compound inhibiting NF-*κ*B activity, increased expression of the inhibitor of NF-*κ*B and reduced total and nuclear expression of NF-*κ*B in endothelial cells. This was associated with reduced oxidative stress, an increase in adiponectin and a reduction in hs-CRP [171, 172]. However, long-term treatment with salsalate and other nonsteroid anti-inflammatory drugs (NSAIDs) has been prohibited due to their increased risk of serious gastrointestinal adverse events, including bleeding, ulceration, and potentially fatal perforation of the stomach or intestines. In addition, an intervention with diclofenac was found to decrease prostaglandin-E2 and TNF-*α* together with a reduction in annexin-A1, caspase-8, and the arachidonic acid metabolite 5,6-dihydroxyeicosatrienoic acid in peripheral blood monocytes [173]. Prolonged therapy with etanercept, which blocks the action of TNF-*α*, improved fasting glucose, increased the ratio of high molecular weight to total adiponectin, and decreased soluble-intercellular-adhesion-molecule- (sICAM-) 1 in obese subjects with abnormal glucose homeostasis and significant subclinical inflammation [174]. However, common side effects were skin reactions at the injection site, a blocked or runny nose, nausea, mild fever, headaches, dizziness, a rash, and stomach problems. Moreover, patients may be more prone to developing infections. Because anti-inflammatory and cell-protective mechanisms mediated by SIRT1 are suppressed in diabetes, small SIRT1 activators are considered to be potential drugs for the treatment of T2DM, obesity, and MetS, among other disorders [175]. However, long-term large-scale clinical studies are lacking.

In spite of these setbacks, however, there are classes of drugs that showed anti-inflammation as a pleiotropic effect. For example, drugs which increased circulating levels of adiponectin such as statins, angiotensin converting enzyme (ACE) inhibitors, and the PPAR activating thiazolidinediones were found to have anti-inflammatory properties [176, 177]. In addition, the glucagon-like peptide-1 agonist exenatide has been used in patients with T2DM to control glycaemia inadequately controlled by metformin alone [178]. In a small study in 24 patients, exenatide-induced glucose lowering was shown to have anti-inflammatory effects independent of weight loss, as evidenced by a reduction in TNF-*α*, IL-1*β*, JNK1, TLR2, TLR4, and SOCS3 in mononuclear cells. This reduction was associated with decreased ROS production [179]. 

Although mechanistically oxidative stress is involved in the development of obesity and associated metabolic and cardiovascular disorders, long-term large-scale studies showing benefit of antioxidant therapy are lacking even more. One of the most studied antioxidants is resveratrol (3,4′,5-trihydroxystilbene), which belongs to a class of polyphenolic compounds called stilbenes [180]. In the test tube, resveratrol effectively scavenges (neutralizes) free radicals and other oxidants [181] and inhibits LDL oxidation [182, 183], associated with inhibition of inflammatory enzymes including cyclooxygenase and LOX [184, 185] by inhibition of proinflammatory transcription factors, such as NF-*κ*B or AP-1 [186, 187]. Resveratrol has also been found to inhibit platelet aggregation [188], to enhance the production of NO [189], and to inhibit inflammatory enzymes [190, 191] *in vivo*. Thus, all these data suggested that resveratrol was a kind of an ideal antioxidant. However, there is little evidence that resveratrol is an important antioxidant in humans [192]. Indeed, a systematic analysis of data obtained until 2011 concluded that “the published evidence is not sufficiently strong to justify a recommendation for the administration of resveratrol to humans, beyond the dose which can be obtained from dietary sources” [193]. A possible explanation may be that upon oral consumption, circulating and intracellular levels of resveratrol in humans are much lower than that of other important antioxidants, such as vitamin C, uric acid, vitamin E, and glutathione. Moreover, the antioxidant activity of resveratrol metabolites, which comprise most of the circulating resveratrol, may be lower than that of resveratrol. Therefore, it was hypothesized that a mixture of dietary products (resveratrol, green tea extract, *α*-tocopherol, vitamin C, n-3 (*ω*-3) polyunsaturated fatty acids, and tomato extract) selected for their evidence-based antioxidant and anti-inflammatory effects may yield more healthy effects than any single molecule. However, a study in overweight men given all these supplements showed only very subtle changes in antioxidant and anti-inflammatory profiles [194]. A possible explanation is that supplementation with dietary antioxidants cannot efficiently overcome the depletion of the antioxidants, which seems to occur faster in obese subjects in agreement with increased oxidative stress [195]. Therefore, antioxidant supplements hardly improve clinical outcomes in these patients [196]. 

One effect of these rather negative results of the former trials may be that our appreciation of the molecular nature of oxidative stress has changed. Indeed, oxidative stress is no longer perceived as a simple imbalance between the production and scavenging of ROS, but as a dysfunction of enzymes involved in ROS production. The focus is on NOXs because they induce the dysfunction of other oxidases including eNOS uncoupling, xanthine oxidase, and mitochondrial dysfunction. Thus NOXs are considered to be important therapeutic targets for drugs such as statins as well as ACE inhibitors, AT1 receptor antagonists (sartans), and aliskiren, as well as spironolactone or eplerenone. Specific NOX inhibitors are under development. However, it remains to be demonstrated that NOX inhibition is more efficient than nonselective scavenging of all ROS by the administration of antioxidants [197, 198].

## 12. Conclusion

**Figure 4:**
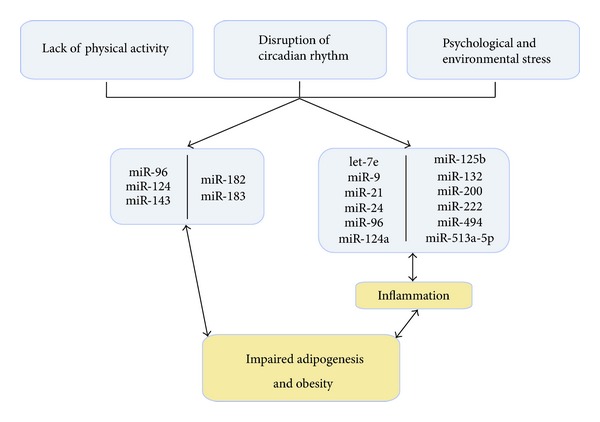
Stress and microRNA expression. A model in which the lack of physical activity, disruption of circadian rhythm, and psychological and environmental stress deregulates the expression of miRs which directly or indirectly through inflammation disrupts adipogenesis and contributes to the development of obesity and associated disorders.

In this paper we showed that obesity is associated with a high risk of metabolic and cardiovascular disorders most probably due to increased inflammation in AT. We discussed the impact of impairment of epigenetic regulatory mechanisms in the development of stress and inflammation. In addition, we reviewed the impact of behaviour and personal and environmental stress on the deregulation of the epigenetic control mechanisms and their possible contribution to the development of obesity and related morbidities. Overall, obese subjects would benefit from a more holistic treatment plan focusing on physiological processes, lifestyle, and environment. In addition, apart from supplementation with antioxidants, novel treatment strategies are required attempting to inhibit tissue oxidative stress. Even when molecules show antioxidant activity *in vitro* and in animal models *in vivo*, long-term clinical trials are needed before it can be concluded that these drugs, such as NOX inhibitors, have an additional therapeutic value. In the near future, inflammation-related miRs, which are commonly deregulated in circulatory and adipose tissue inflammatory cells, may not only be useful as biomarkers for improved risk stratification in obese persons, but may also be considered as therapeutic agents to combat obesity-associated metabolic and cardiovascular diseases. In animal models, disease-inducing cardiac miRs can be persistently silenced *in vivo* through systemic delivery of antimiRs [199]. For example, administration of miR-124 (Figure 4) reduced inflammation by reducing IL6 receptor expression and IL6 secretion [200] and caused deactivation of macrophages [201].
